# A Sigma-Delta ADC for Signal Conditioning IC of Automotive Piezo-Resistive Pressure Sensors with over 80 dB SNR

**DOI:** 10.3390/s18124199

**Published:** 2018-11-30

**Authors:** Behnam Samadpoor Rikan, Sang-Yun Kim, Nabeel Ahmad, Hamed Abbasizadeh, Muhammad Riaz Ur Rehman, Khuram Shehzad, Arash Hejazi, Reza E. Rad, Deeksha Verma, Kang-Yoon Lee

**Affiliations:** 1 Nanoelectronics Group, Department of Informatics, University of Oslo, 0316 Oslo, Norway; behnam@ifi.uio.no; 2 College of Information and Communication Engineering, Sungkyunkwan University, Suwon 16419, Korea; ksy0501@skku.edu (S.-Y.K.); nabeel8@skku.edu (N.A.); Hamed@skku.edu (H.A.); riaz@skku.edu (M.R.U.R.); khuram1698@skku.edu (K.S.); arash@skku.edu (A.H.); Reza@skku.edu (R.E.R.); deeksha27@skku.edu (D.V.)

**Keywords:** Sigma-Delta ADC, flicker noise, pressure sensor, chopper, decimation filter

## Abstract

This paper presents a second-order discrete-time Sigma-Delta (SD) Analog-to-Digital Converter (ADC) with over 80 dB Signal to Noise Ratio (SNR), which is applied in a signal conditioning IC for automotive piezo-resistive pressure sensors. To reduce the flicker noise of the structure, choppers are used in every stage of the high gain amplifiers. Besides, to reduce the required area and power, only the CIC filter structure is adopted as a decimation filter. This filter has a configurable structure that can be applied to different data rates and input signal bandwidths. The proposed ADC was fabricated and measured in a 0.18-µm CMOS process. Due to the application of only a CIC filter, the total active area of the SD-ADC and reference generator is 0.49 mm^2^ where the area of the decimation filter is only 0.075 mm^2^. For the input signal bandwidth of 1.22 kHz, it achieved over 80 dB SNR in a 2.5 MHz sampling frequency while consuming 646 µW power.

## 1. Introduction

**Figure 1:**
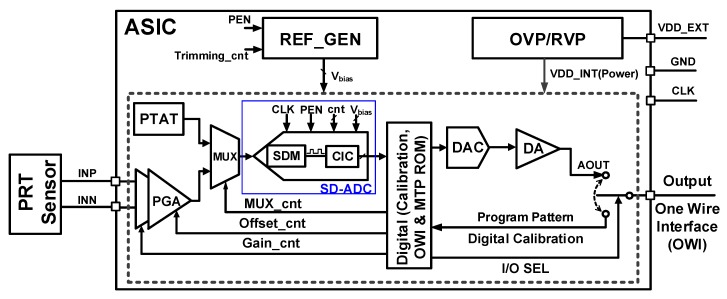
Block diagram of the piezo-resistive type (PRT) signal conditioning IC.

Recently, sensor transducers have been the focus of research for sensor-measuring instruments in automotive and medical systems and have been adopted in pressure transducers and humidity-sensing systems, among others [1,2,3]. Figure 1 presents a monolithic complementary metal-oxide semiconductor (CMOS) auto-compensated sensor transducer for other resistive bridge-type sensors, such as piezo-resistive type (PRT) or strain gauge transducers. The proposed signal conditioning IC is compact and robust to facilitate integration with resistive measuring systems. The signals from the sensors are fed to the signal conditioning IC for low noise amplification, calibration, and signal processing purposes. The presented PRT pressure sensor measures the pressure of the refrigerant of an automotive air conditioner and amplifies the pressure signal information while calibrating the nonlinearities. The electrical output of the sensor should range from 0.5 to 4.5 V when the pressure changes from 0 to 32 bars [4]. The applied PRT sensor provides a signal with the range of 10–110 mV depending on the pressure amount. This is a very small and non-linear signal. Nonlinearity occurs both due to the temperature variation and the nonlinearities of the sensor. At a constant pressure, the temperature variations affect the pressure information. Therefore, the system is calibrated to compensate for temperature variations.

Because the variation of resistance among the sensors differs depending on the pressure, a gauge factor calibration circuit is proposed to compensate for the sensitivity and offset. While the sensor output values indicate the pressure value, the values differ among the sensors. The reliability of the sensor is degraded due to these errors. Thus, the offset, gain, and temperature variation of the resistive pressure sensor must be compensated. Because the resistance of the sensor cannot be directly compensated, the resistance variation of the sensor is converted into an electrical signal and the converted electrical signal is compensated by the Analog Front End (AFE) circuit. As the analog signal is converted to a digital signal, the accuracy in the AFE IC can be improved.

The signal from the PRT is amplified and its range increases to the proper value. This amplification is done by a programmable gain amplifier (PGA). The amplified signal is then fed to the Analog to Digital Converter (ADC) and converted to the digital. Besides, the temperature information of the system is also digitized by the ADC. As it can be observed from Figure 1, the ADC is shared for the temperature and the pressure path signals. The calibration of the system to linearize the pressure information is done digitally. The digital part receives the pressure and the temperature information and calibrates these codes in the way that the temperature variation does not have any effect on the main pressure signal. In addition, it linearizes the nonlinearities of the sensor and gives a linearized signal. Then, the calibrated digital codes are delivered to the Digital-to-Analog Converter (DAC) and by a Drive Amplifier (DA), the output which is a linear signal and independent of the temperature variation is received.

The limitation in the number of the pins and the clock frequency of the system is the reason to convert the calibrated digital code to analog signal again. In this circuit, Proportional to Absolute Temperature (PTAT) is used to provide the temperature information which is converted to digital using ADC. The PTAT circuit output is a voltage range where the minimum voltage value is proportional to the minimum temperature and the maximum voltage value is proportional to the maximum temperature. The operating temperature range of this system is −40 °C to 150 °C. In addition, OVP/RVP is the Over Voltage Protection/Reverse Voltage Protection circuit which protects the over voltages and the reverse voltages to the Application-Specific Integrated Circuit (ASIC). The Reference Generator (REF_GEN) block provides the bias voltages (V_bias_) to the ADC. These bias voltages include V_REFT_, V_REFB_, and V_CM_. Their values are 1.5 V, 0.9 V, and 1.2 V, respectively. Due to the pin limitations of the system, the One Wire Interface (OWI) Communication protocol has been applied in this system to control the registers of the digital part.

The pressure and temperature signals frequencies are low (kHz level). Nevertheless, the precise digitization of these signals is the requirement of the system. Therefore, low speed, high-resolution ADC is required in this system. The expected resolution of this system is over 12-bit. Furthermore, due to the different frequencies of temperature and pressure signals, the signal bandwidth of the ADC, as well as data rate, should be reconfigurable. Moreover, as the structure has been designed to have small size, and as the frequency and the Least Significant Bit (LSB) values of the signals are small, the flicker noise issue is a concern of the ADC in this structure. The Successive Approximation Register (SAR) ADC as a candidate is reported as the most power efficient ADC structure. Nevertheless, as the resolution of the SAR ADCs are increased, these structures become bulky and the mismatch problem in the DAC would be more serious [5,6,7].

This paper presents a second order discrete-time Sigma-Delta (SD)-ADC with over 80 dB SNR designed for signal conditioning IC for the automotive piezo-resistive pressure sensors. The SD-ADC consists of Sigma-Delta Modulator (SDM) and Cascaded Integrator Comb (CIC) filter. The CIC is used as the decimation filter and it has reconfigurable structure to control the data rate of the SD-ADC. The main challenge of this work is to keep the area of the SD-ADC below 0.5 mm^2^, while keeping the resolution over 12-bit. Therefore, this structure avoids applying Half-Band (HB) filters in the decimation filter as they are bulky and power-hungry blocks [8,9,10,11]. Consequently, a small area SD-ADC is proposed in this paper. Furthermore, the noise performance in the SDM has been improved due to choppers applied in the integrators. Therefore, the flicker noise is not the limiting factor of the resolution in the applied structure.

The remainder of this paper is organized as follows. Section 2 discusses the design and implementation of the SDM and its sub-blocks; Section 3 presents and describes the CIC as the decimation filter; Section 4 demonstrates the post-simulation and measurement results; and finally, the paper is concluded in Section 5.

## 2. Architecture of the SDM

**Figure 2:**
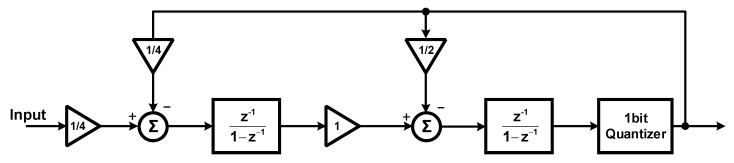
Block diagram of the Sigma-Delta Modulator (SDM).

**Figure 3:**
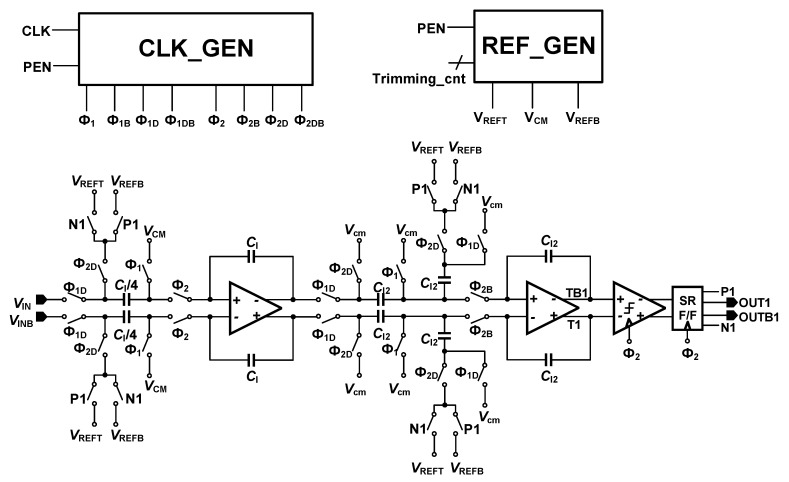
Circuit implementation of the SDM, including Reference and Clock Generators (CLK_GEN).

Figure 2 presents the block diagram of the SDM. This is a second order discrete-time SDM with Cascaded Integrator Feed-Back (CIFB) structure. The required resolution of the system is just 12 bits; to keep the area of the SD-ADC below 0.5 mm^2^, only second-order SDM is used. Generally, the low order SDMs show better stability performance. The system applies 2.5 MHz clock frequency. The required bandwidth of the system is at kilohertz level. Therefore, the Over-Sampling Ratio (OSR) is selected to be 1024 and 2048. This is a reconfigurable structure and the OSR values can be applied based on the input frequency bandwidth, the clock frequency, and the decimation factor of the CIC filter. The reason the SD-ADC needs to be configurable is that the pressure and the temperature signals have different frequencies, but they share the same ADC. Therefore, the system has been designed to have a configurable structure and able to control the input signal bandwidth and data rate. The modulator loop is the cascade of two switched capacitor integrators, where the output is fed back to the integrators with the coefficients of 0.25 and 0.5. These coefficients are the ratio of the sampling and feedback capacitors. These values have been derived from MATLAB/Simulink modeling and justified with the Cadence Spectre simulations. This structure uses a comparator as a 1-bit quantizer. The circuit implementation of the SDM is presented in Figure 3. The differential input signals are sampled in the sampling capacitors, as shown in Figure 3. The ratio of the sampling capacitor (C_I_/4) and the capacitor around the amplifier (C_I_) decides the feedback coefficient values of the first integrator. The switch sizes are 10 µm/0.18 µm and the C_I_ value in Figure 3 is 20 pF. The gain bandwidth of the amplifiers is designed to be over 5 times of the sampling frequency. By proper sequencing of the switches and applying non-overlap control signals, channel charge-injection effects can be neglected. Besides, to design the parasitic insensitive integrators, adopting proper clock timing to the sampling capacitors is essential [12].

**Figure 4:**
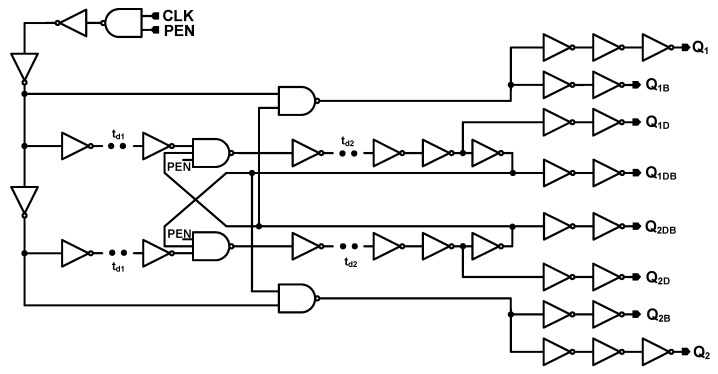
Structure of the clock generator.

**Figure 5:**
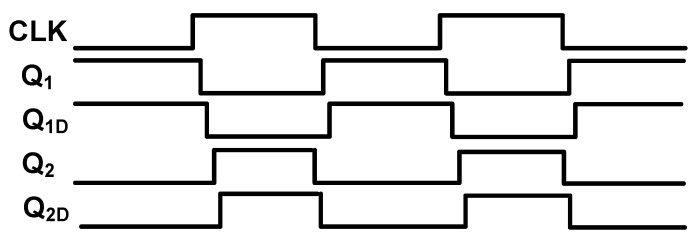
Clock signals generated on the clock generator block.

The structure of the clock generator (CLK_GEN) is shown in Figure 4. The Q_1_, Q_1D_, Q_2_, and Q_2D_ clock signals are generated through this circuit. However, as the switches in Figure 3 are TGATEs—which apply both PMOS and NMOS transistor switches, Q_1B_, Q_1DB_, Q_2B_, and Q_2DB_—clock signals that are the inverted signals of the Q_1_, Q_1D_, Q_2_, and Q_2D_ are also generated on clock generator circuit. It is notable that Q_1D_ and Q_2D_ are delayed form of Q_1_ and Q_2_ signals. Figure 5 shows the clock signals generated on the clock generator block. The Q_1B_, Q_1DB_, Q_2B_, and Q_2DB_ signals are not shown on this figure as they are just the inverted form of Q_1_, Q_1D_, Q_2_, and Q_2D_ signals. As it can be observed, Q_1_ and Q_2_ signals are non-overlapped clocks, as well as Q_1D_ and Q_2D_ signals. It is notable that to be visible, the delays in Figure 5 are exaggerated. This block was designed for worse cases and with some margins to avoid the delays differences due to process variations. The PEN signal is controlled by the OWI which is included in the Digital block in Figure 1. The clock signal (CLK) is applied to this circuit externally.

**Figure 6:**
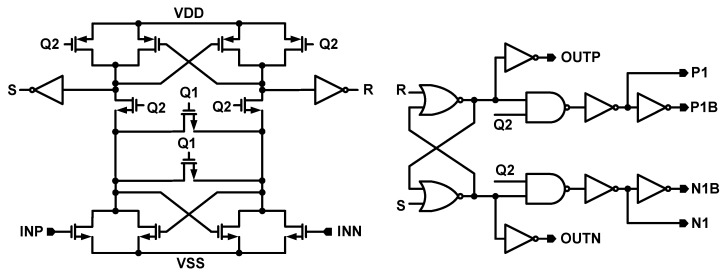
Structure of the comparator.

The structure of the comparator is presented in Figure 6. The dynamic type comparator is used in this structure, as it has lower power consumption comparing to the static ones [13,14]. The differential outputs of the second integrator are connected to the INP and INN inputs of the comparator. The outputs of the comparator (OUTP and OUTN) are decided through latches. Q_1_ and Q_2_ clock signals control the comparator clocking and the P1 and N1 signals (Figure 6) are decided and used to control some of the switches in the SDM as it is shown in Figure 3.

**Figure 7:**
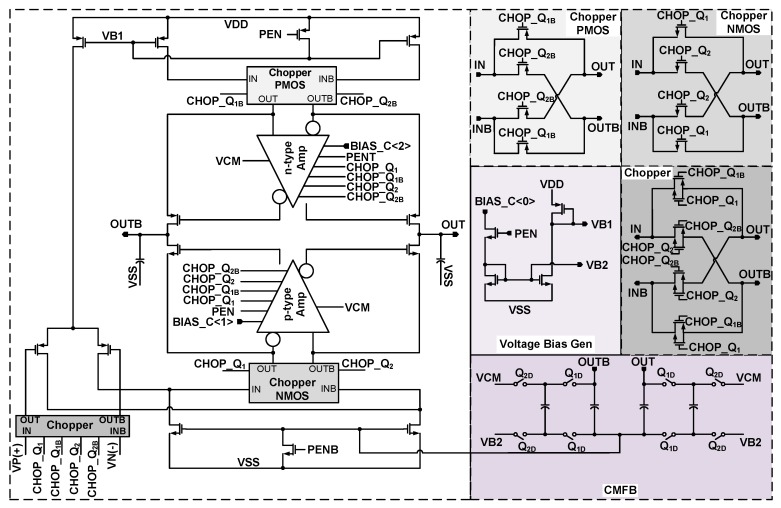
Structure of the applied gain-boosted amplifier.

**Figure 8:**
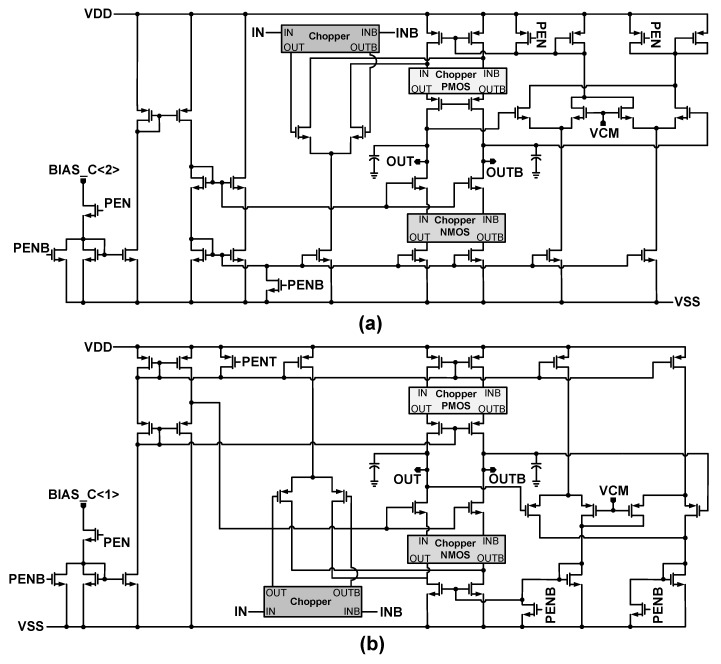
Structure of the gain-boosting amplifiers (**a**) n-type and (**b**) p-type.

Figure 7 shows the structure of the amplifier which is applied to the integrator. The amplifier has gain-boosted p-type Folded-Cascode structure. This structure is slightly different than the gain-boosted amplifier presented in [15]. The chopping has been applied to the inputs of the gain boosting amplifiers as well. The DC gain of this amplifier is over 100 dB. The gain boosting amplifiers adopt n-type and p-type Folded-Cascode structures as they are shown in Figure 8a,b. The choppers are used in these amplifiers to reduce the effect of flicker noise in the lower frequencies. The choppers clock frequencies are decided in the way that the flicker noise is pushed to the frequencies that are out of the band of interest. According to where these choppers are applied, they adopt p-type (Chopper PMOS), n-type (Chopper NMOS), or p- and n-type (Chopper) MOS transistors as their switches. The structures of these choppers are shown in Figure 7. To reduce the flicker noise in low frequencies, the choppers are applied to the gain-boosting amplifiers as well, as shown in Figure 8. All the designed amplifiers apply the common mode feedback (CMFB) in their circuitry to keep the output bias voltage levels. The applied choppers in Figure 8 are like that of Figure 7 and are not repeated in Figure 8. Furthermore, their clock signals are also not shown in this figure. As it is shown, the choppers apply clocks in their circuitry. These clock signals are shown as CHOP_Q_1_, CHOP_Q_1B_, etc., in Figure 7. The clock generator to produce these clocks is similar to Figure 4. Nevertheless, the frequency of these clocks is lower than the sampling clock. In addition, it is notable that to make the design and layout easier, the amplifiers in the first and second integrators are designed to be the same. Nevertheless, the chopping in each of the amplifiers can be enabled or disabled.

## 3. Configurable Decimation Filter

In SDM design, as the order of the modulator increases, the slope of the noise shaping also increases. Therefore, due to the increment of the slope of the noise shaping, higher order decimation filter is required. This results in high order, high power, and large area filters to be cascaded. For low power and high-resolution requirements, such as IoT and Multichannel, the use of lower order modulators and multiplier-less filters are desirable [16].

**Figure 9:**
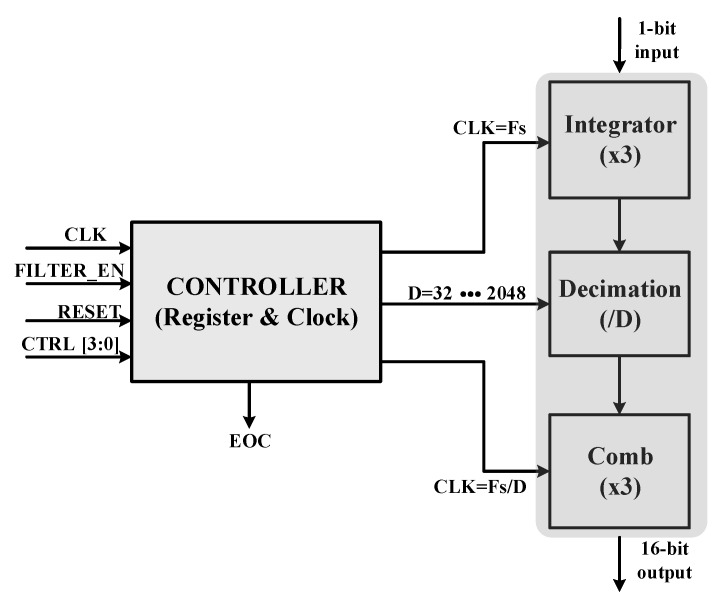
Block diagram of the decimation filter.

The target resolution of the designed SD-ADC is over 12-bit. The SDM has second order configuration, as mentioned before. In order to achieve the desired resolution, a sinc3 filter is adopted for this design. The decimation filter is a CIC filter [17], with a configurable option for different data rates, as shown in Figure 9. It is composed of three integrators and three comb filters in cascaded form with a controller. The cascaded integrator and the comb filter implements the transfer function (Equation (1)) and (Equation (2)) [18]. It means that the decimation factor can be changed to adjust ADC output for different input bandwidth. Therefore, resultant ADC can be used for a variety of applications by easily changing the data rate at the filter stage.

(1) $$H\left(z\right)=H_{\mathrm{comb}}^{N}\left(z\right)*H_{\mathrm{integrator}}^{N}\left(z\right)$$

(2) $$H\left(z\right)=\frac{1}{M^{k}}{\left(\frac{1-z^{-M}}{1-z^{-1}}\right)}^{k}$$

As shown in Figure 9, a controller is added to the conventional CIC filter. This controller is used to select the register width and proper clocking within the filter stages. Integrator clock is the same as sampling clock (Fs) whereas the comb filter operates as the ratio of the sampling clock to the decimation factor (D) (Fs/D). This reduces the power consumption at the combing stage of the CIC filter. This clock is handled by a controller as per requirements of the decimation factor.

Register size is varied through the controller when the decimation factor is changed. It is due to the fact that integration and differentiation (comb) needs different register sizes when the decimation factor is changed. Register sizes are directly proportional to the decimation factor and increases when decimation factor is increased. They are related by the following equation [19]:

(3) $$\mathrm{World}\;\mathrm{Length}=L\left(\log_{2}D.M\right)+W_{\mathrm{in}}$$

where L is the number of stages in CIC filter (3 here), D is decimation factor (variable), M is the number of delays (M = 1), and W_in_ is the input word length (2 bits after 2’s complement). Register size is controlled inside the integrator and comb filter for the specific decimation factor. The output of the filter is the 14 Most Significant Bits (MSB), which are selected from the single wide register (main register). Remaining LSBs are discarded.

**Figure 10:**
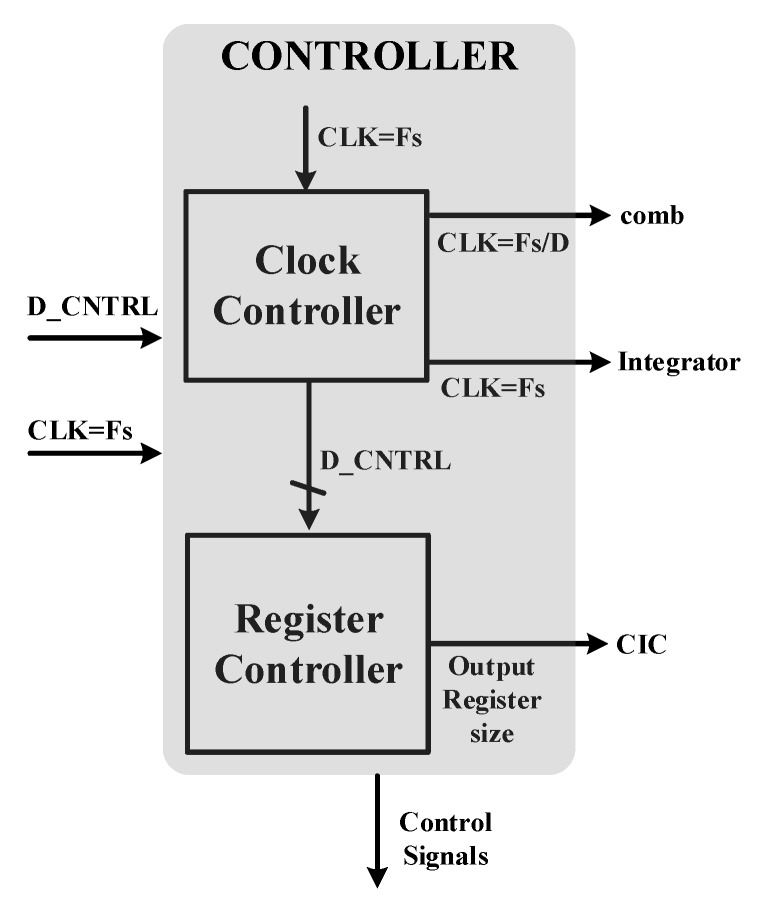
Controller for configurable CIC filter.

The controller used in this architecture is comprised of a clock controller and a register controller, as shown in Figure 10. The clock controller applies the necessary clocks required for all blocks. Furthermore, it generates some control signals required for the measurement setup. Register size can be calculated from Equation (3) and largest possible size (for D = 2048) yields to be 35 bits for an ideal noise shaping case. This will be the main register used by all filters. The controller will select MSB 14-bits from this register depending upon the decimation factor and the remaining bits will be discarded.

The controller has the clock input of Fs (sampling clock) that is used by SDM as well. This clock is used to generate the necessary clocks for integrator, comb and other control blocks. D_CNTRL (4 bits) are used to select among various options of decimation factors (32, 64, …, 2048). This signal is controlled by OWI. Register controller block selects 14 MSBs from the main register and assigns to the output by discarding the LSBs.

## 4. Experimental and Post-Simulation Results

**Figure 11:**
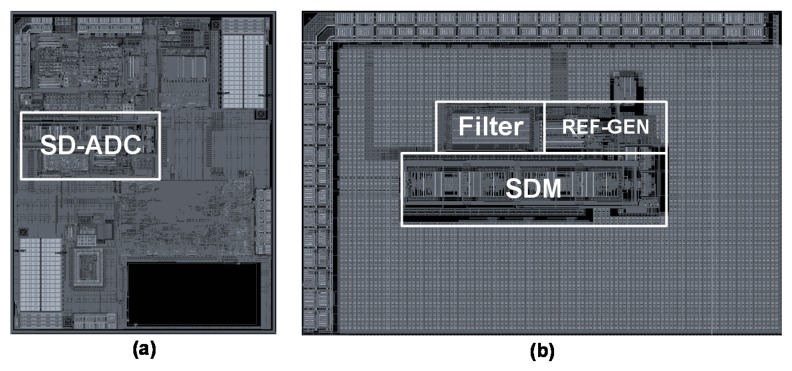
Layout of (**a**) the signal conditioning IC of the automotive piezo-resistive pressure sensor (**b**) the Sigma-Delta (SD) Analog-to-Digital Converter (ADC) in a separate chip for measurement purposes.

The prototype SD-ADC was designed and fabricated in a 180 nm CMOS process with one poly and four metal layers. The total active area of this SD-ADC including SDM, decimation filter, and reference generator (REF-GEN) is 1075 µm × 460 µm. Figure 11a presents the layout of the designed signal conditioning IC of the automotive piezo-resistive pressure sensor, which includes SD-ADC. For measurement purposes, the SD-ADC is also fabricated in a separate chip which is shown in Figure 11b.

**Figure 12:**
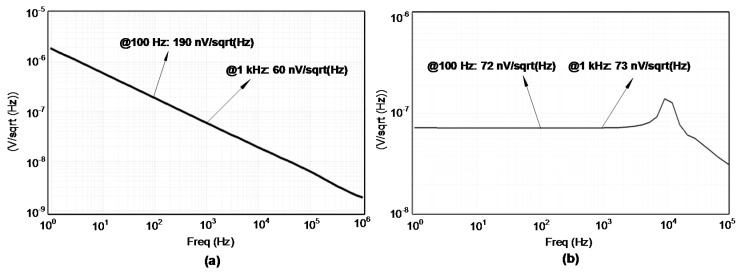
Noise performance of the SDM in post-simulation (**a**) without chopper and (**b**) with chopper.

Figure 12 shows the noise simulation of the SDM in log-log scale. Figure 12a shows the noise of the system when chopper is not applied. For the low frequencies, flicker or 1/f noise is the dominant noise while for the higher frequencies over the corner frequency, white noise is the dominant source of the noise. Integrating the noise from DC would have a high value for this case. This is the post-simulation result that includes the R, C, and Coupling Capacitance (CC) parasitic extracted from the layout. The noise at 100 Hz is 190 nV/sqrt (Hz) and at 1 kHz is 60 nV/sqrt (Hz). Figure 12b shows the noise of the system when the chopper is applied. As we can observe with the chopper, the flicker noise has been reduced. This is also the post-simulation result, which includes the R, C, and CC parasitic extracted from the layout. For this case, the noise at 100 Hz is 72 nV/sqrt (Hz) and at 1 kHz is 73 nV/sqrt (Hz). The integration of the noise in the bandwidth of the interest, which is from DC to 1.22 kHz, is much less than the LSB value of the SDM. For this simulation, the chopping clock has been selected to be 10 kHz.

**Figure 13:**
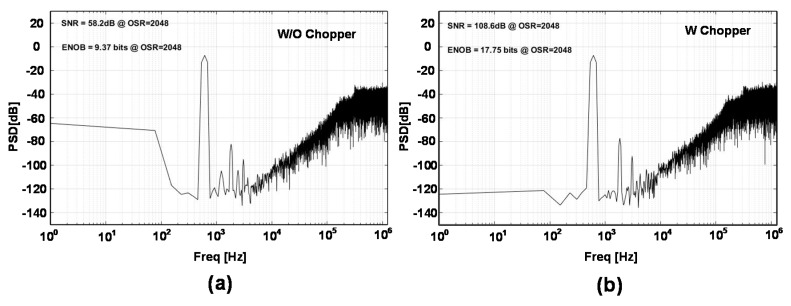
Post-simulation performance of the SDM for F_S_ = 2.5 MS/s, OSR = 2048, 2^15^ number of samples and rectangular windowing (**a**) without chopper and (**b**) with chopper.

The experiment in Figure 13 compares the performance of SDM with and without chopping. These are also the post-simulation results of the SDM which include the R, C, and CC parasitic extracted from the layout. The output of the SDM which is a 1-bit data series has been extracted from the post simulation and has been analyzed and plotted in MATLAB using rectangular window. To be able to observe the effect of flicker noise better, the input signal range as small as 100 mV has been applied for these simulations where the LSB value is less than noise. As can be seen in Figure 13a, the low-frequency noise is dominant. To remove this noise, the chopping has been applied in Figure 13b and the performance of the structure has been improved and the lower frequency noise level has been reduced. The chopping frequency in Figure 13b is 10 kHz like Figure 12b. The number of samples for both simulations are 2^15^. It is notable that the resolution of the SDM is 17.75 bit which is much higher than required resolution for the PRT system. However, the limiting factor of the resolution in the SD-ADC is in decimation filter where to reduce the area, only CIC filter has been applied and HB filters have been skipped for the design.

**Figure 14:**
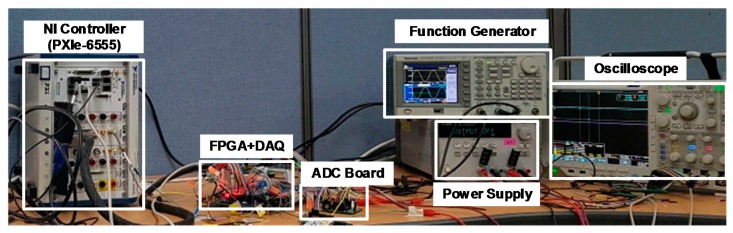
Measurement environment.

Figure 14 shows the measurement environment. Oscilloscope, Function Generator, LabVIEW, FPGA, and Power Supply are applied in this measurement. Function Generator can generate the sine wave for AC analysis and ramp signal for the DC analysis. Nevertheless, for high-resolution ADCs a signal with high SNR is required which can be generated from LabVIEW. The Power Supply generates the DC voltages and Oscilloscope is used to monitor the signals. The digital codes are analyzed in LabVIEW and FPGA is applied for the clock and control signals synchronization.

**Figure 15:**
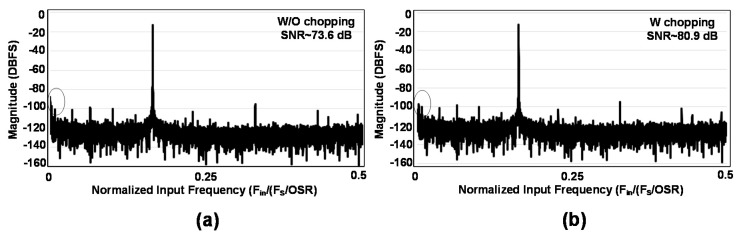
Measurement results of the FFT spectrum for 0.4 kHz sine wave sampled at 2.5 MS/s with OSR of 1024, 2^14^ number of samples and Hanning window (**a**) without chopping and (**b**) with chopping.

The FFT spectrum of the measured SD-ADC is shown in Figure 15. Figure 15a shows the spectrum without chopping where the noise at lower frequencies is notable. Applying chopper in Figure 15b has improved the SNR by around 7 dB. For these measurements, OSR is selected to be 1024, the sampling frequency is 2.5 MHz, and the chopping frequency is 10 kHz. The input frequency is 400 Hz and the input signal range is 300 mV. The Signal to Noise and Distortion Ratio (SNDR) for the measurement in Figure 15b is over 80 dB and the Effective Number of Bits (ENOB) is over 13-bits. The ENOB for the input signals from DC to FS/(2 × OSR) remains over 12.6-bits. The Hanning window has been applied for these measurements and the number of samples are 2^14^. Besides the harmonics, some other tones exist in the spectrums. The reason is that the application of the CIC filter cannot completely attenuate the out of band noise, especially around Nyquist frequencies; thus, these tones have been aliased back in the band of interest. Nevertheless, as far as these tones are lower than the harmonics and the ENOB is over 12-bits, we neglect them in trade off with lower area and power consumption for the applied decimation filter. Application of the half-band filters in the decimation filter would have improved the ENOB and would have reduced such tones but at the cost of higher area and power consumption.

**Figure 16:**
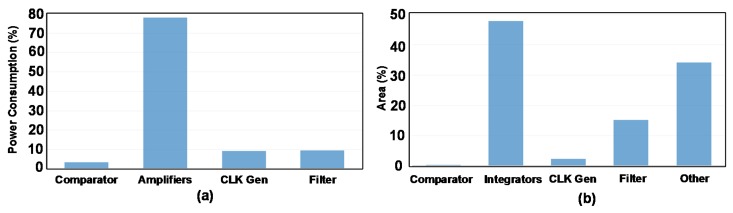
(**a**) Power consumption break-down and (**b**) area consumption break-down.

The total power consumption of the SD-ADC is about 646 µW from a 2.4 V supply voltage. The power break-down is shown in Figure 16a. As it can be seen, around 78% of the power is consumed in amplifiers while this portion for the filter, CLK_GEN, and comparator are 10%, 9%, and 3%, respectively. The area break-down is also presented in Figure 16b. Integrators with 48%, consume the largest area of this structure. The filter occupies only 15% of the area. In this figure, “Other” relates to current and voltage generator, the digital part of the chip to control the trimmings and testability, and dummies.

Finally, Table 1 summarizes the performance of the SD-ADC, including SDM and decimation filter, and compares it with other ADC structures. The work in [20] has a lower area but at the same time lower ENOB. Although the work in [21] shows a higher resolution, it has a larger area. The proposed structure has a similar area as [22] and [23]. However, it shows better ENOB performance when compared to these structures.

Table 2 compares the decimation filter with other works [24,25,26]. For the area comparison which is the contribution of this work, decimation filters with similar performances has been selected. As can be observed, this structure has a lower area and power consumption when compared to the others. Table 3 compares the SDM with other works [27,28,29,30,31]. When considering area comparison, the structures with comparable performances have been selected. The area that is the concern of this work is much smaller than [27,29] and is comparable to [30,31], although the latter uses a 90 nm technology. The input noise density performance is also summarized in Table 2. Due to chopping, it shows better input noise density performance than other structures that have reported the noise performance. The designed SDM has a higher resolution when compared to other works reported in this table.

## 5. Conclusions

In this paper, a second-order discrete-time SD-ADC was presented. The structure has been applied in a signal conditioning IC for automotive piezo-resistive pressure sensors. To improve the noise performance of the structure, the choppers have been adopted in every stage of the amplifiers of the integrators. Applying chopper reduces the flicker noise of the structure. Besides, the area and power of the filter were reduced by adopting only CIC as the decimation filter and skipping the usage of the half-band filters, which is common in the decimation filters. The filter had a reconfigurable structure that was able to be applied to different data rates. This was accomplished by applying a controller in the CIC filter that was used to select the register width and proper clocking within the filter stages. The proposed ADC was fabricated and measured in a 0.18-µm CMOS process. Due to the application of a CIC filter and the avoidance of using HB filters, the active area of the SD-ADC is 0.49 mm^2^, where the area of the decimation filter is only 0.075 mm^2^. The ADC achieved the SNDR of 80.9 dB in a 2.5-MHz sampling frequency and 1.22 kHz input signal bandwidth, while consuming 646 µW from a 2.4-V power supply according to the measurement results.

## Figures and Tables

**Table 1:** Performance summary of SD-ADC and comparison.

	This Work	[20]	[21]	[22]	[23]
Process (nm)	180	180	180	180	180
Sample Rate (MS/s)	2.5	0.5	10.24	0.32	5
ENOB (bits)	>12.6	10.94 *	14.73 *	11.82	10.76
Power (mW)	0.646	0.291	12.99	0.031	0.49
OSR	1024	-	256	40	–
Fin (kHz)	1.22 for OSR = 1024	250	20	4	2500
Area (mm^2^)	1.075 × 0.460	0.655 × 0.472	0.98 × 0.903	0.5	0.48

* Simulation results.

**Table 2:** Decimation filter comparison.

	This Work	[24]	[25]	[26]
Process (nm)	180	130	180	350
Input no. of Bits	1	3	1	6
Decimation Factor	32–2048	32–128	128	16–32
SNR (dB)	>78	74	>100	>96
Area (mm^2^)	0.075	0.211	9	19
Power Consumption (mW)	0.064	1.5	0.9	-
Data Rate (kHz)	1.22–78.125	9750	128	230–460

**Table 3:** SDM comparison.

	This Work	[27]	[28]	[29]	[30]	[31]
Process (nm)	180	130	40	180	180	90
Input Stage	PMOS	–	PMOS	PMOS and NMOS	PMOS	–
Input Noise Density (nV/√Hz)	73 @ 1kHz	–	140	–	98	–
±Input Range (mV)	300	–	50	–	40	–
Supply Voltage (V)	2.4	1.4	1.2	0.9	1.8	1.2
Area (mm^2^)	0.236	0.44	0.06	1.8	0.2	0.17
ENOB (bit)	17.75	12.06	12.14	14.05	12	10.2
Input Bandwidth (kHz)	1.22	2000	2	20	2	10,000
FOM (pJ/Conv.step)	1.05	0.43	1.16	0.15	7.69	0.575
FOM (dB)	171.8	168.0	154.7	173.8	148.1	128.7
